# Testing a Computerized Cognitive Training Protocol in Adults Aging With HIV-Associated Neurocognitive Disorders: Randomized Controlled Trial Rationale and Protocol

**DOI:** 10.2196/resprot.6625

**Published:** 2017-04-26

**Authors:** David Vance, Pariya Fazeli, John Shacka, William Nicholson, Peggy McKie, James Raper, Andres Azuero, Virginia Wadley, Karlene Ball

**Affiliations:** ^1^ School of Nursing University of Alabama at Birmingham Birmingham, AL United States; ^2^ Department of Pharmacology & Toxicology University of Alabama at Birmingham Birmingham, AL United States; ^3^ School of Medicine University of Alabama at Birmingham Birmingham, AL United States; ^4^ Department of Psychology University of Alabama at Birmingham Birmingham, AL United States

**Keywords:** cognitive aging, cognitive remediation therapy, cognition therapy, HIV associated cognitive motor complex

## Abstract

**Background:**

HIV-associated neurocognitive disorders occur in nearly 50% of adults with HIV. Such disorders can interfere with everyday functioning such as driving and medication adherence. Therefore, cognitive interventions are needed to address such neurocognitive disorders as well as improve everyday functioning, especially as people age with HIV.

**Objective:**

This article reports and discusses the overall rationale and development of speed of processing training, a computerized Internet cognitive training program, to improve this specific neurocognitive ability as well as everyday functioning and quality of life in adults aging with HIV. Although this protocol has been shown to improve speed of processing, everyday functioning, and quality of life in healthy, community-dwelling older adults in the advanced cognitive training in vital elderly (ACTIVE) study, its efficacy in adults aging with HIV has not been established. Nevertheless, such a cognitive intervention is particularly germane as 52%-59% of adults with HIV experience HIV-associated neurocognitive disorders (HAND), and both the frequency and severity of such disorders may increase with advancing age.

**Methods:**

The description of this longitudinal randomized controlled trial covers the following: (1) rationale for speed of processing training in this clinical population, (2) overview of overall study design, (3) eligibility criteria and HAND, (4) intervention dosage, (5) assessment battery, and (6) examination of biomarkers.

**Results:**

The project was funded in April 2016 and enrolment is on-going. The first results are expected to be submitted for publication in 2020.

**Conclusions:**

Similar novel cognitive intervention approaches are suggested as they may be of value to those with HAND and may utilize similar features of this current randomized controlled trial (RCT) protocol to examine their therapeutic efficacy.

**Trial Registration:**

ClinicalTrials.gov NCT02758093; https://clinicaltrials.gov/ct2/show/NCT02758093 (Archived by Webcite at http://www.webcitation.org/6p8C5fBCX)

## Introduction

Combination antiretroviral therapy (cART) has resulted in improved health-related outcomes and longer life in adults with human immunodeficiency virus (HIV), with some studies estimating length of life being tantamount to those without HIV [1]. Despite such optimism, neuroinflammation, substance abuse, stress, stigma, depression and anxiety, and poorer educational quality often associated with HIV may contribute to other poorer health-related outcomes such as neurocognitive impairment, often referred to as HIV-associated neurocogntiive disorders (HAND) [2]. HAND is an objective diagnosis determined by administration of a neurocognitive assessment measuring at least seven neurocognitive domains (eg, speed of processing, verbal memory). With such an assessment, when patients score greater than 1 standard deviation below their age and education norm on 2 or more neurocognitive domains, they are considered to have HAND; this classification rubric is referred to as the Frascati criteria [3]. Using the Frascati criteria, 52% to 59% of adults with HIV experience HAND [4]. Furthermore, there are gradations of HAND of increasing severity in terms of the neurocognitive and everyday functioning impairments, with ~33% experiencing asymptomatic neurocognitive impairment, ~12% experiencing mild neurocognitive disorder, ~5% experiencing mixed neurocognitive disorder, and ~2% experiencing HIV-associated dementia [5].

With such well documented neurocognitive impairments, neurocognitive aging in this group represents a major concern since by 2020, 70% of adults with HIV in the United States will be 50 years and older [6,7]. Thus, there is a growing population that is particularly vulnerable to HAND due to the cooccurrence with aging-related neurocognitive impairments as well as age-related comorbidities that likewise compromise brain health. For example, in a study of 162 older (50+ years) and younger (<50 years) adults with and without HIV, Vance and colleagues [8] found as a group, older adults experienced more neurocognitive impairments. Such neurocognitive impairments affect driving safety, medication adherence, instrumental activities of daily living, and quality of life [9-13]. Furthermore, in the cART era, these neurocognitive impairments continue to be observed in several domains including memory, executive functioning, and 1 area of particular importance—speed of processing [14-29].

Speed of processing is the rate at which neurocognitive functions are performed [30-32]. People with HIV are vulnerable to speed of processing declines [33,34], especially as they age [30-32]. In a meta-analysis of 41 HIV neurocognitive studies from both the pre- and post-cART era [33], speed of processing was among the neurocognitive domains demonstrating the greatest decline from early to late stages of HIV for all ages. More recent studies also show that speed of processing deficits are common and persist in the post-cART era [14-29]. In fact, a 2014 study of 186 adults with HIV found that speed of processing “fully mediated the effects of age on learning, memory, and executive functioning and partially mediated the effect of major depressive disorder on learning and memory” (p. 806) [35] while other HIV studies show speed of processing deficits impair real-world functioning [13,36]. Such speed of processing declines are associated with poorer driving performance and more at-fault crashes in healthy older adults [30,37,38] as well as middle-aged (40+ years) and older adults with HIV [10,13,39,40], which is a growing public health concern [10,13,39]. In the Southern United States, specifically in the Deep South, these points are highly relevant because (1) even with speed of processing declines, adults with HIV must rely on their own driving, especially in rural areas with limited public transportation; and (2) the epicenter of HIV has emerged here in the last decade [41,42], which means many adults with lower socioeconomic status backgrounds and African Americans with HIV will also have HAND [43,44]. Few behavioral interventions have aimed to improve neurocognition in this vulnerable population [45], and pharmacological cognitive interventions produce adverse side effects in a population already experiencing multiple comorbidities [45-50].

Fortunately, some types of computerized cognitive interventions have been shown to improve neurocognition without adverse side-effects [51-53]. Despite the known efficacy of computerized cognitive training programs, only 2 such studies have examined this in adults with HIV. One study attempted to improve global neurocognition in a mixed sample of 30 adults with and 30 adults without HIV; unfortunately, only 54% (25/46) of those assigned to the active condition were able to use the system successfully and probably as a result, no therapeutic neurocognitive benefit was derived [54]. Yet, in 1 study involving adults aged 40+ years with HIV, Vance and colleagues [55] randomly assigned 22 to receive 10 hours of speed of processing training and 24 to receive a no-contact control condition. Compared with the no-contact control group, those who received the speed of processing training improved significantly on a measure of visual speed of processing called the useful field of view (UFOV) test as well as on the timed instrumental activities of daily living test, which is a laboratory measure of everyday functioning. Related to everyday functioning, a subsequent cross-sectional driving simulator study by Vance and colleagues [13] also demonstrated that in 26 adults aged 40+ years with HIV, poorer visual speed of processing was predictive of poorer driving ability (eg, average gross reaction time, divided attention reaction time). In fact, more self-reported automobile accidents in the previous 2 years were associated with slower gross reaction time and a higher number of collisions in the driving simulator. Although this was not a cognitive training study, the results suggest that improving speed of processing may likewise improve driving in aging adults with HIV.

Targeting an intervention that specifically improves speed of processing has both theoretical and neurocognitive appeal. According to the diminished speed of processing theory [32,56-59], the rate at which adults mentally process information slows with age. Speed of processing declines can occur at all stages of processing, from the speed at which information is encoded to the execution of a response [60,61]. This reduction in speed of processing places demands on other neurocognitive systems [31]. For example, Lindenberger et al [62] found that age-related decrements in memory, reasoning, and fluency were all mediated through differences in speed of processing. A subsequent study showed that increased age affects speed of processing (effect size=−0.69) to a greater degree than memory (effect size=−0.25), or executive functioning or reasoning (effect size=−0.27), thus reinforcing the focus on speed of processing [63]. Furthermore, electrophysiological studies already indicate that adults who receive speed of processing training, compared with controls, experience increased N2pc and P3b amplitudes (electrical signals detected on the scalp), which is reflective of capacity enhancement and attentional allocation [64]. The lack of attentional and inhibition control associated with prefrontal dysregulation, especially in HIV [65,66], may be an inefficient way to process information quickly and accurately. Thus, speed of processing training may reduce dependence on frontally-oriented activity by reallocating such responses to such posterior brain regions which can improve speed of processing and in turn translate to everyday functional improvements [64,67].

Based on findings from these earlier studies, this study was proposed and funded by the National Institute of Mental Health (1R01MH106366-01A1 – “An RCT of Speed of Processing Training in Middle-Aged and Older Adults with HIV”). This randomized controlled trial (RCT) study described in this article examines a well-documented intervention in the neurocognitive and gerontological literature called speed of processing training (a cognitive remediation therapy) in the population of aging adults with HIV. Specifically, there are 3 study aims: To (1) examine whether 10 versus 20 hours of speed of processing training provides differential therapeutic responses on improving this neurocognitive ability over time; (2) examine whether 10 versus 20 hours of speed of processing training will differ in therapeutic value on improving everyday functioning (ie, IADLs, driving) over time; and (3) examine whether improvement in speed of processing and everyday functioning over time mediate improvement in quality of life indices (eg, depression, locus of control, health-related quality of life). In doing so, these 6 key areas of the study are described: (1) rationale for speed of processing training in this clinical population, (2) overview of study design, (3) eligibilty criteria and HAND, (4) intervention dosage versus control condition, (5) assessment battery, and (6) assessment of biomarkers. Finally, the complexity of this research design is examined in relation to other cognitive interventions.

## Methods

### Rationale of Speed of Processing Training

Several computerized cognitive training programs have been examined in the normal geriatric population; some focus on improving functioning in a particular neurocognitive domain such as executive functioning or memory, whereas others attempt to improve more global neurocognitive functioning. Albeit, given the resources of time and effort required to engage in training programs, selecting which neurocognitive domain to be “improved” must be chosen judiciously. Whereas some experts and clinicians may focus on memory training given its salience to noticeable memory complaints, others have found targeting other domains to have more long-lasting effects that likewise produce improvements in other areas in which training was not targeted.

In the advanced cognitive training in vital elderly (ACTIVE) study, researchers from 6 sites across the United States randomized normal, community-dwelling older adults (65+ years; N=2802) to 1 of the following treatment arms: (1) speed of processing training, (2) memory training, (3) reasoning training, and (4) no-contact control. After just 10 hours of training, those in the speed of processing training group experienced significant improvements in this neurocognitive ability. In fact, the National Institute on Nursing Research and the National Institute on Aging (January 14, 2014) announced that speed of processing training used in the ACTIVE study enabled “older people to maintain their cognitive abilities as they age,” even 10 years after training [68]. Furthermore, in a meta-analysis of 52 computerized cognitive training studies, Lampit and colleagues [52] found that treatment effect sizes varied widely depending on what neurocognitive domain was being targeted. No significant effect sizes were observed for cognitive training that targeted executive functioning or attention, whereas statistically significant small to moderate effect sizes were observed for verbal memory (g=0.08), working memory (g=0.22), nonverbal memory (g=0.24), and visuospatial skills (g=0.30), with the most robust finding observed for speed of processing training (g=0.31). These data suggest that this particular neurocognitive domain may be more amenable than others for improvement during neurorehabilitation.

To promote successful aging and optimal functioning in the aging HIV population, speed of processing is a preferred target for this cognitive training intervention based on the following points. First, speed of processing is 1 of the most essential neurocognitive abilities that declines with aging, beginning in one’s 40s [69-71]. With HIV considered as a form of accelerated aging [72,73], concerns about more profound speed of processing declines in this population increase. Second, speed of processing declines are related to poorer everyday functioning (eg, driving, performing IADLS) as well as lower quality of life (eg, health-related quality of life) [74]. Third, speed of processing training has been shown to improve this neurocognitive ability [75,76]. Fourth, this improvement in speed of processing has been shown to translate into improved driving performance, mobility, and performance on IADLs [77-83]. Fifth, in community-dwelling older adults, these neurocognitive improvements (ie, using a speed of processing measure called useful field of view) have been shown to be robust over several years; such long-term improvements may also be produced in adults with HIV. Finally, in the ACTIVE study, additional beneficial outcomes have been identified as a result of speed of processing training that include: (1) improved self-rated health [84], internal locus of control [85,86], and health-related quality of life [87-89]; and (2) protection against depression [90]. These outcomes reflect areas that must be addressed in adults with HIV who also may have reduced health-related quality of life [91-93], poor self-rated health [94], decreased locus of control [92,95-97], and depression [45,91,97,98]. These quality of life outcomes are essential areas in HIV that likewise require intervention [99]. This RCT of 264 adults with HAND extends the ability to demonstrate whether speed of processing training can improve speed of processing and everyday functioning not only in the short-term, but also during an extended 2-year period.

### Overview of Overall Study Design

**Figure 1:**
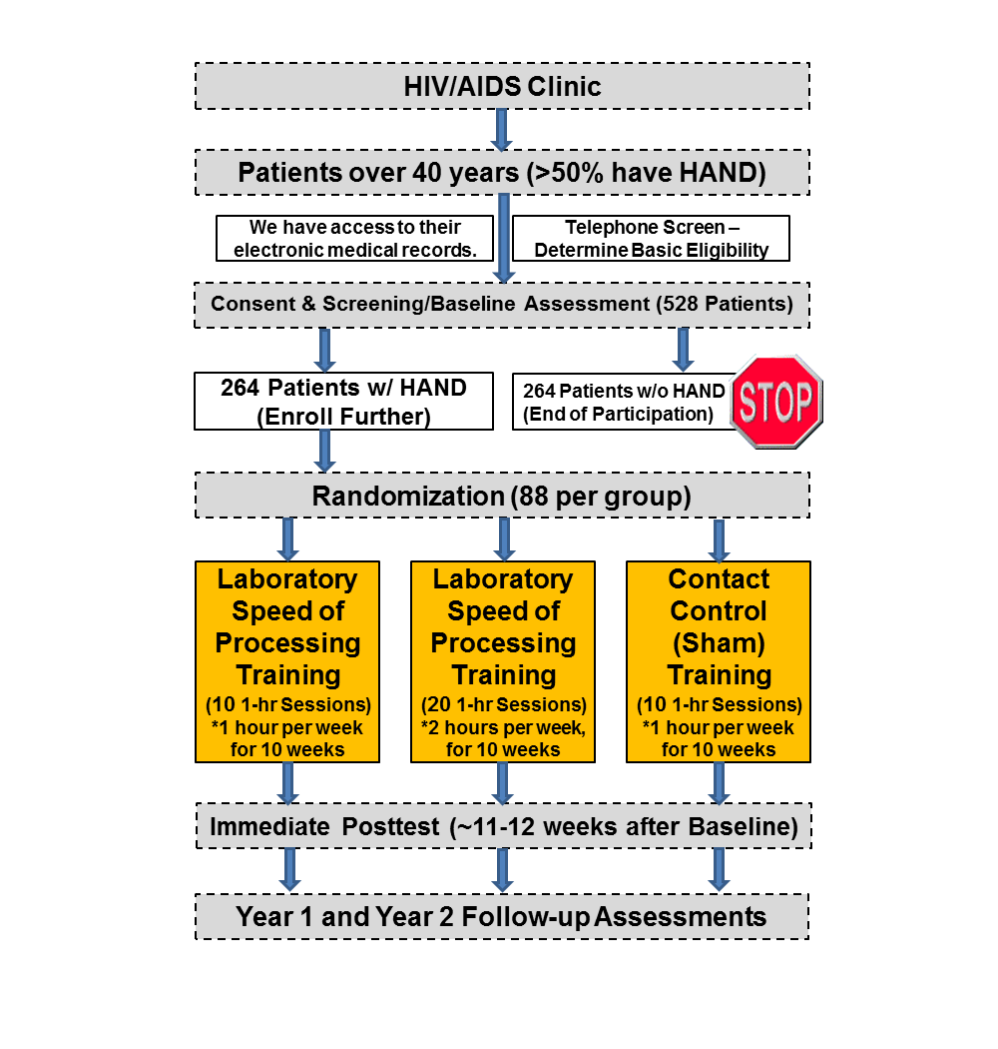
Overall study design flowchart.

A pre-post 3-group experimental longitudinal design is used (Figure 1). Participants with HAND are administered a neurocognitive, functional, and quality of life assessment battery at baseline, approximately 11-12 weeks postintervention and annually for up to 2 years. This translational science study recruits adults 40+ years with HAND and assigns them to 1 of 3 groups: (1) 10 hours of speed of processing training, (2) 20 hours of speed of processing training, and (3) 10 hours of Internet navigation training (ie, a contact-control condition).

Recruitment flyers are posted at a university HIV and acquired immunodeficiency syndrome (AIDS) clinic targeting those 40+ years only. Interested potential participants call the study telephone number to be told more about the study and to determine if they meet basic eligibility requirements (eg, HIV+, 40+ years); if they do, they are scheduled for a baseline assessment appointment. During this appointment, participants are consented and administered a neurocognitive, everyday functioning, and quality of life assessment. From this assessment, a HAND diagnosis is determined. Based on prior prevalence rates, it is expected over half of the participants experience HAND; only those with HAND and thus who need such a cognitive intervention are randomly assigned to 1 of the 3 treatment arms. Block stratified random assignment will minimize the risk of imbalance among randomized groups in 2 key factors: minority status and speed of processing deficiency measured by a cut off using the useful field of view test [100]. Completion of each arm should take participants approximately 10 weeks. After training, participants are administered the same baseline assessment at posttest and then annually for 2 years. Two years of follow-up is justified because it is needed to determine the robustness of the speed of processing training over time in this clinical population as observed in the older adults in the ACTIVE study. To retain participants over a 2-year period, several strategies are employed; here are a few examples: (1) sending reminder holiday and birthday cards to participants; (2) providing little gifts to participants with the study name, phone number, and logo on them; and (3) gathering information on secondary contacts of people they know so we can track them down in case such participants move. In addition, participants are compensated for their time they spend with the study.

### Recruitment Protocol, Rationale, and Targeting HAND

Eligibility criteria specifically focus on casting a wide net to ensure that the study findings are generalizable to the larger HIV population. Albeit, given the focus on driving and neurocognition, certain inclusion, exclusion, and HAND criteria were chosen.

#### Inclusion Criteria

Since driving-related factors are being examined as 1 of the outcomes of the intervention, participants must be licensed drivers when entering the study. Otherwise, participants (men and women) must be 40+ years, English-speaking, and have HAND.

#### Exclusion Criteria

Due to this study being longitudinal, participants not living in stable housing (eg, halfway house) are excluded because of the challenge of scheduling follow-up visits. Potential participants are excluded if they indicate that they are planning to move away from the Birmingham metropolitan area within the next 2 years. Furthermore, participants with significant neuromedical comorbidities (eg, schizophrenia, epilepsy, bipolar disorder, multiple sclerosis, Alzheimer disease or related dementias, mental retardation) are excluded; such major neurological comorbidities may confound study results and interfere with the cognitive training. These comorbidities are assessed in the telephone screen and confirmed after the baseline assessment using the university HIV and AIDS Clinic electronic medical charts. Other conditions (eg, legally blind or deaf [vision confirmed at baseline], currently undergoing radiation or chemotherapy, a history of brain trauma with a loss of consciousness greater than 30 minutes) that could impact neurocognitive functioning or testing also necessitates exclusion; again, this information is conferred in the telephone screen and again at baseline. These typical exclusion criteria are used in many HIV neurocognitive studies [12,14,101-104]; we wish to exclude those with other major neurological conditions besides HIV that affect neurocognition.

### HIV-Associated Neurocognitive Disorders

This study focuses on HAND and speed of processing training. Typically, speed of processing declines emerge in one’s 40s [70] and perhaps even earlier for those with HIV, which likely contributes to HAND [73]. In fact, the prevalence of HAND may be greater in middle-aged and older adults with HIV [12,18,105,106]. Primary reasons for the focus on HAND are: (1) participants have room to improve neurocognitively from speed of processing training (ie, no ceiling effects), and (2) a cognitive intervention for HAND is needed. Using a neurocognitive battery that measures performance in several neurocognitive domains, the Frascati criteria is used to determine HAND [104]. If a participant’s neurocognitive scores are greater than 1 standard deviation below demographically-adjusted means in at least two neurocognitive domains (eg, memory, speed of processing), they are determined to have HAND. Further breakdown of the type of HAND diagnosis can be made based upon functional impairment too (ie, Lawton and Brody activities of daily living) to compare differences in treatment response for those with varying severity of HAND. In consensus, 2 doctorally-trained psychologists determine HAND based upon the demographically adjusted *t* scores.

### Intervention Dosage Versus Control Condition

Both the speed of processing and contact-control training protocols require participants to visit the laboratory to engage in 1-hour sessions with passive supervision from a research assistant. The research assistant greets participants, helps them log onto the computer, answers questions participants may have about the training, and monitors the time participants engage in their assigned treatment arm.

In the speed of processing training protocol, participants are trained to improve the speed or accuracy in which they identify and locate visual-spatial information using 5 games or exercises from the POSIT Science, which has been used in our prior studies. The games include: (1) Sweep Seeker (fundamental speed of processing), (2) Bird Safari (visual accuracy), (3) Target Tracker (multiple object tracking), (4) Master Gardener (eye movements), and (5) Double Decision (UFOV). These games are automatically customized to the participants’ individual ability. The speed, difficulty, and complexity of each game is systematically increased as participants successfully master specified performance criteria. Manipulations used to increase difficulty include decreasing the duration for which the visual stimuli are presented, adding visual or auditory distracters, increasing similarity between targets or distracters, and presenting visual targets over a broader spatial expanse, which expands one’s (UFOV) and is important for driving [107].

Studies using speed of processing training often vary from 10 to 20 hours. As mentioned in the prior study in adults with HIV, 10 hours of training was sufficient to produce significant improvement in UFOV and everyday functioning in the short-term. The NIH-funded ACTIVE study (N=2802 normal older adults) also initially used 10 hours of speed of processing training, but additional “booster” training was found to improve the effect size and these improvements were robust over several years [108]. A meta-analysis [52] of cognitive training in older adults found specifically for speed of processing training, a dose of 20 hours or less produced a significantly higher effect size (0.34) compared with more than 20 hours (0.24). Given these dosage considerations, in this study 1 treatment arm receives 10 hours of training and 1 treatment arm receives 20 hours of training, whereas the control arm of the study will receive 10 hours of nontherapeutic computer contact. This approach will allow our study to determine the optimal therapeutic dosage over time. Likewise, conclusions from this meta-analysis [52] suggest that optimal effect sizes from speed of processing training for our study will be observed when training sessions are at 60 minutes and administered 1-3 times per week—dosage parameters already incorporated in this study.

In the contact-control group, participants receive 10 hours of Internet navigation training; it has been used successfully as an optimal social or computer contact-control condition for speed of processing training studies. This contact-control (sham) condition mirrors speed of processing training with the same amount of social contact with study staff and computer exposure, but does not provide any therapeutic neurocognitive benefit as we have previously observed [75,81,109]. Specifically, participants are given instructional materials and exercises on how to navigate the Internet. For more computer savvy participants, they are directed to other websites that may be of interest. These Internet activities reflect those which people do normally and do not have any observable neurocognitive therapeutic effect. This approach has been used in prior National Institute on Aging-sponsored studies [75]. This contact-control group is being used to compare with the other intervention in which only 10 hours of the speed of processing training are being provided; this will allow for a direct comparison. Meanwhile, the intervention with 20 hours of training is being included to test whether the extra dosage will be more effective versus 10 hours of speed of processing training alone.

Finally, a treatment fidelity checklist is used so staff can review with participants the amount of time that they have engaged in training. Furthermore, the POSIT Science software monitors the amount of time participants spend engaged in each exercise. As in the ACTIVE study, participants are considered to be trained when they successfully complete 80% of the training [107]. This completer-only analysis is appropriate for use when examining the actual potential of the speed of processing training. If participants do not complete training, their data can be examined using an intent-to-treat analysis.

## Results

The project was funded in April 2016 and enrolment is on-going. The first results are expected to be submitted for publication in 2020. Study measures are assessed at baseline, immediate posttest following training, and at 2 annual follow-ups. These assessments are categorized by: (1) demographic, background, and covariate measures; (2) Aim 1, neurocognitive measures; (3) Aim 2, everyday functioning measures; (4) Aim 3, quality of life measures (see Table 1). Most of these measures use standardized instruments that have good to excellent psychometric properties; this is particularly relevant for the neurocognitive assessments as they are used to determine HAND [12,55,110-118].

**Table 1:** General domains assessed overtime.

Demographic, background, and covariate measures	Aim 1: Neurocognitive measures	Aim 2: Everyday functioning measures	Aim 3: Quality of life measures
Demographic questionnaire Wide range achivement test- 4 (Educational quality) [119] Drug urine screen HIV history and status Electronic medical records	Speed of processing (ie, UFOV^b^) [120] Attention and working memory (PASAT^a^-2000) [121] Learning ie, Hopkins verbal learning test- Revised) [115,116,122] Verbal memory (ie, Hopkins verbal learning test- Revised) [115,116,122] Verbal fluency (ie, controlled oral work association test) [123] Executive function (ie, Wisconscin card sorting test) Psychomotor (ie, grooved pegboard) Reported instrumental activities of daily living (IADLs) (ie, Lawton & Brody activities of daily living questionnaire)	Driving simulator [13,82,107] Driving habits questionnaire [124] Retrospective & prospective state crash records [125] Timed instrumental activities of daily living [126,127] Medication aherence	Centers for epidemiological studies- Depression (CES-D) [128,129] Internal locus of control [85] Self-rated health and health related quality of life (measured via medical outcomes study short-form (SF-36) [130] Neurocognitive Complaints

^a^PASAT: Paced auditory serial attention test.

^b^UFOV: Useful field of view.

One primary and novel focal area of this study (Aim 2) is driving and driving-related outcomes. Most adults with HIV experience some degree of neurocognitive impairment, and coupled with the lack of adequate public transportation or social support, many adults must rely on their own driving and navigational skills to carry out basic IADLs such as grocery shopping and visiting their medical providers. Thus, this cognitive intervention is expected to benefit such driving outcomes [77,131-135].

Driving is assessed in 3 primary ways: (1) driving simulation, (2) the driving habits questionnaire, and (3) retrospective and prospective state crash records [124]. As mentioned earlier, the driving simulator has been used in earlier studies to document the relationship between functioning in certain neurocognitive domains on particular driving simulator outcomes [13]. Such standardized, performance-based outcomes include: average gross reaction time, percentage of time driving outside of the lane, percentage of time driving over the posted speed limit, and so on.

The driving habits questionnaire [124] is a self-report survey that assesses driving exposure and driving avoidance across varying difficult driving circumstances (eg, driving at night). Driving exposure [124] is assessed with 4 self-reported items: (1) number of days per week driven (0–7), (2) miles driven per week (numeric estimate), (3) miles driven per year (estimated to the nearest 2500 miles), and (4) driving space (the further one has driven from home in the past year). These items can then be standardized (*z*-scored) and summed to form a driving exposure composite score.

Driving avoidance [124] is assessed with 10 self-reported items that ascertain whether participants passed up opportunities to drive in the past 3-month period due to avoiding difficult driving circumstances: (1) driving at night, (2) driving in bad weather, (3) driving alone, (4) driving on interstate highways or expressways, (5) driving in unfamilar places, (6) driving on high traffic roads not including interstates, (7) driving in rush-hour traffic, (8) making lane changes, (9) making left-hand turns across oncoming traffic, and (10) merging into traffic while entering a highway or expressway. Likewise, these items can then be standardized (*z*-scored) and summed to form a driving exposure composite score.

Retrospective and prospective vehicle state crash records are also accessed through the Alabama Department of Transportation (ADOT). As in past studies, using participants’ driver’s license numbers, a request is made to the ADOT office requesting for accident reports which are publically available records and made available for a small fee. These files contain police reports that describe the circumstances of the accident. Based upon these reports, a determination of whether participants were at fault or not is determined by 2 independent raters; when there is a lack of an agreement, a third rater makes the final determination. Thus, these state crash records can be used to determine what variables are correlated to the number of total crashes, number of at-fault crashes, and number of not-at-fault crashes. The use of actual, real life vehicle crash records provides convergent validity to the other driving measures and is also ecologically valid.

Crash records can be examined both retrospectively and prospectively. Retrospectively, once the entire initial sample’s baseline data are collected, participants’ driving records can be requested for the 5 years prior to starting our study and examined to determine crash rates and neurocognitive predictors of crashes. Prospectively, once participants have completed the training in our 2-year study, it can be determined through logistic regression and hierarchical regression whether this intervention was effective in reducing the frequency and severity of vehicle crashes. Given the infrequent nature of vehicular crashes, the large sample size of this study will provide insight into whether this intervention is effective in helping adults with HAND maintain safe driving. Although obtaining information on participants’ driving exposure and driving avoidance may modify the risk of crashes for some of our participants, their combined influence can be examined over time as these variables are assessed at each time point.

## Discussion

### Examination of Biomarkers

A unique featue of this study is the incorporation of biomarkers of brain health and brain chemistry through a separate K99 and R00 (K99AG048762) grant mechanism that uses the infrastructure of the parent R01 to examine the relationship between such biomarkers on neurocognitive and everyday functioning. In the neuroscience literature, it is clear that even basic biomarkers such as stress hormones or the amount of HIV virus in the blood can impair certain cognitive functions [136]; thus, measuring such biomarkers may help examine their impact on cognition. And since such biomarkers are known to impact cognitive functioning, the presence of such biomarkers may either facilitate or hinder the training effects of the intervention. Furthermore, studies also show that when people or organisms are exposed to novel stimuli and learning situations, these environmental stimuli can change the neurochemistry of the brain that can be detected through blood draws [137-139]. Thus, these biomarkers are derived from 2 sources. First, since all the participants are recruited from an internal HIV and AIDS clinic, their most current as well as future physiological lab values (eg, triglyceride levels, glucose levels, CD4+ lymphocyte count, HIV viral load) is easily accessed through the clinic’s medical database. Logistically, an added feature of this internal access is that the study does not have to expend additional resources of time, money, and participant burden to acquire such information. These basic biomarkers are relevant as they may affect neurocognitive functioning [18,24,140,141] as well as influence (or be influenced by) training gains from the intervention (ie, if a participant is experiencing cognitive problems from elevated triglycerides, then his poorer cognitive functioning may hinder how well he benefits from the training protocol).

Second, blood draws from a subset of participants (approximately 200 who are first to agree to be in this substudy) during the time of the baseline visit are also conducted to collect aliquots of blood to test for various inflammatory biomarkers (eg, IL-6, souble CD14) and neurotrophic factors (eg, insulin-like growth factor), changes in many of which have been associated previously with HAND and HIV(+) patients with neurocognitive decline [142-145]. Again, monitoring these specific biomarkers is equally relevant because they may not only affect neurocognitive functioning [136] but they may also influence training gains from the intervention, and be influenced by the intervention. As mentioned earlier, exposure to novel stimuli has been shown to change brain chemistry[138,139]; thus, participating in this intervention may likewise change brain chemistry which may be detected with blood draws and looking for certain biomarkers. Moreover, positive correlations in changes of these candidate blood biomarkers with respect to our training intervention may suggest their future utility to clinicians for monitoring disease progression, and response to therapy.

### Conclusions

This study protocol examining a cognitive remediation program is reflective of other methodologies that examine their benefits over time. As such, this study design lends itself to other types of computerized cognitive remediation programs that are online and utilize computer gaming features to improve particular neurocognitive abilities [75,107]. Other cognitive remediation programs are touted to improve executive functioning, language, memory, attention, and even improve social functioning skills and reduce rumination (eg, cognitive bias modification) [146,147]. This protocol can be easily modified to examine the efficacy of these programs. In fact, with enough resources, these programs can be examined side-by-side and in combination to examine their efficacy over time. Furthermore, other biomarkers and brain imaging techniques could also be applied to examine how these cognitive remediation programs specifically are influenced and even alter brain chemistry and brain morphology [53,148]. Future studies may even examine the influence of such computerized cognitive remediation protocols in delaying or mitigating the effects of disease-related and age-related neurocognitive declines, mild neurocognitive impairment, and dementia.

## Abbreviations

ACTIVE: advanced congnitive training in vital elderly
AIDS: acquired immunodeficiency syndrome
cART: combination antiretroviral therapy
HAND: HIV-associated neurocognitive disorders
HIV: human immunodeficiency virus
RCT: randomized controlled trial

## Appendix

Multimedia Appendix 1 NIH peer-review feedback.
