# Supplementary material for: Testing a Computerized Cognitive Training Protocol in Adults Aging With HIV-Associated Neurocognitive Disorders: Randomized Controlled Trial Rationale and Protocol
Source: JMIR Res Protoc. 2017 Apr 26;6(4):e68. doi: 10.2196/resprot.6625 (PMC5422019; doi:10.2196/resprot.6625)
Supplement: Multimedia Appendix 1 [file resprot_v6i4e68_app1.PDF]

SUMMARY STATEMENT
(Privileged Communication)

PROGRAM CONTACT:
Pim Brouwers
240-627-3863
ebrouwer@mail.nih.gov

Release Date: 11/15/2015

Application Number: 1 R01 MH106366-01A1

Principal Investigator

VANCE, DAVID E PHD

Applicant Organization: UNIVERSITY OF ALABAMA AT BIRMINGHAM

Review Group: BSCH
Behavioral and Social Consequences of HIV/AIDS Study Section
AIDS - EXP. REV.

Meeting Date: 11/05/2015
Council: JAN 2016
Requested Start: 04/01/2016

RFA/PA: PA14-127
PCC: 9A-ASPC
Dual IC(s): AI

Project Title: An RCT of Speed of Processing Training in Middle-Aged and Older Adults with HIV

SRG Action: Impact Score: 21 Percentile: 10
Next Steps: Visit http://grants.nih.gov/grants/next_steps.htm
Human Subjects: 30-Human subjects involved - Certified, no SRG concerns
Animal Subjects: 10-No live vertebrate animals involved for competing appl.
Gender: 1A-Both genders, scientifically acceptable
Minority: 1A-Minorities and non-minorities, scientifically acceptable
Children: 3A-No children included, scientifically acceptable
Clinical Research - not NIH-defined Phase III Trial

Project Year	Direct Costs Requested	Estimated Total Cost
1	411,903	605,497
2	475,140	698,456
3	409,499	601,963
4	320,875	471,686
5	332,064	488,134
TOTAL	1,949,481	2,865,736

ADMINISTRATIVE BUDGET NOTE: The budget shown is the requested budget and has not been adjusted to reflect any recommendations made by reviewers. If an award is planned, the costs will be calculated by Institute grants management staff based on the recommendations outlined below in the COMMITTEE BUDGET RECOMMENDATIONS section.

NEW INVESTIGATOR

1R01MH106366-01A1 VANCE, DAVID

NEW INVESTIGATOR

RESUME AND SUMMARY OF DISCUSSION: This application proposes to examine the effects of speed of processing training (10 vs. 20 hours of laboratory-based training vs. contact controls) in 264 HIV+ persons with HIV-Associated Neurocognitive Disorder who are over 40. Speed of processing training has been shown to have a lasting impact in older adults and the proposed intervention has the potential to improve the ability of HIV+ persons with HAND to perform their instrumental and functional activities of daily living. Therefore, the proposed study has the potential to be of high public health impact. The investigative team is strong and the application is highly responsive to concerns raised in the prior review. The home-based training arm has been dropped and now two different dosages of the training are compared. Some remaining minor weaknesses in the study were discussed and it was noted that these could be addressed using data that will be collected. As a result, the committee's overall enthusiasm for this much improved resubmission was high.

DESCRIPTION (provided by applicant): As people age with HIV, the synergistic effects with normal age-related cognitive declines will accentuate and/or accelerate declines in cognitive functioning which can be detected as early as in one's 40s. Although interventions are needed to protect/improve cognitive functioning, one intervention already exists to improve speed of processing. NINR/NIA (January 14, 2014) announced that Speed of Processing Training used in the ACTIVE Study (N = 2,802 community-dwelling older adults) has the ability to enable "older people to maintain their cognitive abilities as they age" even 10 years after training. As shown in the ACTIVE Study, this intervention uniquely improves driving, instrumental activities of daily living (IADL), health-related quality of life, self-rated health, internal locus of control, and protects one from depression; these represent areas of needed intervention for adults with HIV as well. In adults with HIV, our pilot studies likewise indicate speed of processing declines are associated with poorer driving simulator performance and more self-reported at-fault automobile crashes; such speed of processing declines on driving alone represent a significant public health concern. We also demonstrated that Speed of Processing Training improved this cognitive ability and translated into improved performance on a timed measure of IADLs. Based on our prior research, this RCT proposal consists of a pre-post two-year longitudinal experimental design whereby 264 adults with HIV, 40+ years and diagnosed with HIV-Associated Neurocognitive Disorder, will be randomly assigned to one of three training conditions: 1) 10 hours of laboratory-based Speed of Processing Training, 2) 20 hours of laboratory- based Speed of Processing Training, or 3) 10 hours of a standardized computer-contact control (sham) condition. AIM 1: Determine whether 10 vs 20 hours of speed of processing training will improve this cognitive ability at post-test, year 1, and year 2 after baseline. AIM 2: Determine whether 10 vs 20 hours of speed of processing training will improve everyday functioning at post-test, year 1, and year 2 after baseline. Exploratory AIM: Determine whether improvement in speed of processing and/or everyday functioning over time mediate improvement in quality of life (e.g., depression).

PUBLIC HEALTH RELEVANCE: Cognitive aging with HIV is concerning due to three facts: 1) using Fascati criteria, ~50% of adults with HIV experience HIV-associated Neurocognitive Disorders (HAND), 2) by 2020, 70% of those with HIV will be 50 and older, and 3) age-related cognitive declines may exacerbate HAND. Such cognitive impairments occur in a number of domains (i.e., memory, attention) that negatively impact everyday functioning and quality of life. Fortunately, as observed in the 10-year ACTIVE Study (N = 2,802) which examined memory, reasoning, and speed of processing training, speed of processing training has superior properties to improve this cognitive ability and translate to improvements in everyday functioning and quality of life which has been shown in our pilot studies to

improve outcomes for middle-aged (40+) and older adults with HIV; this study will build upon this existing science to improve cognitive and everyday functioning in adults with HIV.

CRITIQUE 1:

Significance: 1
Investigator(s): 1
Innovation: 2
Approach: 2
Environment: 1

Overall Impact: In this highly responsive resubmission, the investigators continue to propose a trial of a well-established cognitive training intervention in HIV-infected older adults. Given the evidence for continuing cognitive impairments in those with HIV infection in spite of effective antiretroviral treatments and the lack of other effective interventions to improve cognitive and functional status in these persons, the proposed study has high significance and potential public health impact. The researchers have addressed previous concerns about intervention dosage, format (eliminating a difficult to understand home-based training arm) and underlying conceptual model, making this application have high potential overall impact.

1. Significance:

Strengths

- As noted above, this application would address a highly significant issue related to cognitive and functional impairments in HIV-infected individuals.
- The inclusion of daily functional status and driving skills makes the proposed trial have even greater practical significance.

Weaknesses

- None

2. Investigator(s):

Strengths

- The team is strong, with extensive experience in cognitive training interventions, aging and cognition, and working with persons with HIV infection.
- The team also has extensive experience in behavioral intervention trials.

Weaknesses

- None

3. Innovation:

Strengths

- Although similar studies have been done with other populations, this would be the first significantly trial of this cognitive training strategy with older individuals with HIV infection.

Weaknesses

- None

4. Approach:

Strengths

- This continues to be a strong application from an experienced team with clear expertise in the proposed intervention strategy.
- The change from a home-based arm to two arms with different doses of cognitive training is a significant strength.

Weaknesses

- Although the active intervention is presented as representing primarily speed of processing training, several of the Posit training activities have different goals (e.g., jewel diver has a significant working memory component and Bird Safari focuses on visual attention).
- Although the investigators indicate that participants will have HAND based on Frascati criteria, it is not clear from the tables of measures and the text how this diagnosis will be established. The Frascati criteria include subjective cognitive problems and it's not clear how they will be assessed.
- While the team allows for attrition in the planned data analyses, it seems likely that some participants will not complete either the full 10 or 20 hours within a given time period. Will participants continue until they complete all hours of training (no matter how many weeks) or will they be dropped after a given period of time?

5. Environment:

Strengths

- The environment is very strong, with clear clinical and research resources that will support the execution of the study.

Weaknesses

- None

Protections for Human Subjects:

Acceptable Risks and/or Adequate Protections

Data and Safety Monitoring Plan (Applicable for Clinical Trials Only):

Acceptable

Inclusion of Women, Minorities and Children::

- Sex/Gender: Distribution justified scientifically
- Race/Ethnicity: Distribution justified scientifically
- Inclusion/Exclusion of Children under 21: Excluding ages < 21 justified scientifically
- Participants will be 40 years of age or older

Vertebrate Animals:

Not Applicable (No Vertebrate Animals)

Biohazards:

Not Applicable (No Biohazards)

Resource Sharing Plans:

Acceptable

Budget and Period of Support:

Recommend as Requested

CRITIQUE 2:

Significance: 2
Investigator(s): 1
Innovation: 2
Approach: 4
Environment: 1

Overall Impact: Combination antiretroviral therapy (cART) for HIV has transformed it into a chronic disease that may span several decades of life. As people with HIV age, the synergistic effects of normal age-related cognitive decline and HIV-related neurocognitive disorders (HAND) become more relevant. This resubmission application proposes to examine the effects of speed of processing (SOP) training on 264 HIV+ adults with the goal of determining whether training will improve cognitive ability and everyday functioning at post-test, 1 and 2 years after training. The investigative team is strong and well-experienced. Preliminary work by the applicants shows promise of SOP training to improve cognitive performance in middle aged adults with HIV. Enthusiasm is diminished by a number of methodological limitations, such as potentially confounding effects of gender, substance use disorders, and different CNS penetrance of various cART medications.

1. Significance:

Strengths

- Cognitive aging among HIV+ individuals is a major concern
- Both HIV and aging are associated with declines in speed of processing that are related to driving deficits, which will become even more pronounced as the HIV population ages
- This research group developed the SOP training program, one of the only two computerized cognitive interventions conducted in adults with HIV. SOP training has been shown to protect against depression and improve quality of life in older adults

Weaknesses

- Though the applicants argue that speed of processing deficits are common and persist in the post-HAART era, the majority of the evidence that they provide is based on pre-cART studies

that are over 10 years old, when the most common deficits did indeed involve speed of processing. More recent reviews not included in the application (e.g. Heaton et al. 2011) indicate that the most common pre-cART deficits were in motor skills and cognitive speed, whereas cART deficits are predominantly in memory and executive function.

2. Investigator(s):

Strengths

- The PI is an established and very productive investigator with extensive expertise in the fields of HIV, cognitive aging, and cognitive interventions
- Strong investigative team

Weaknesses

- None noted

3. Innovation:

Strengths

- Few non-invasive approaches to improve cognition have been attempted in adults with HIV and HAND.

Weaknesses

- Most of the literature and methods appear somewhat dated and not particularly innovative

4. Approach:

Strengths

- The research team has extensive experience with SOP training

Weaknesses

- The only pilot data with SOP training of HIV+ individuals showed effects on processing speed 5 weeks post training. What reason do the applicants have to believe that the training will have long lasting effects that will be measurable 1 and 2 years post training? Their pilot data with older HIV- adults shows that the effects of the training seem to dissipate with time (2 years post-training).
- There is evidence that cART with relatively high CNS penetrance have detrimental effects on cognitive functioning relative to cART with lower CNS penetrance, particularly on procedural tasks such as the ones proposed. No consideration has been given to the CNS penetration effectiveness of different cART medications, which may confound the speed of processing results.
- The neurocognitive battery requires 1.5 hours of administration. The purpose of including a neurocognitive battery of significant length (1.5 hours) is not entirely clear, as the proposal provides conflicting information: on one hand it is noted that the battery is included solely for determination of HAND, whereas later it is noted that all of the 7 neurocognitive domains assessed will be used as outcome measures. There are no specific hypotheses related to neurocognitive function (other than for speed of processing) and the inclusion of these measures needs to be better justified given the increase in subject burden that it introduces and the additional statistical comparisons that it entails.

- The HIV literature reveals significant sex differences in cognitive function that are particularly pronounced on procedural tasks such as the ones in the proposed study, on which women have been reported to be more impaired. It is concerning that the effects of gender have not been taken into account.
- Individuals with substance use disorders will be included, which may have a confounding effect.

5. Environment:

Strengths

- Excellent

Weaknesses

- None

Protections for Human Subjects:

Acceptable Risks and/or Adequate Protections

Data and Safety Monitoring Plan (Applicable for Clinical Trials Only):

Acceptable

Inclusion of Women, Minorities and Children:

- Sex/Gender: Distribution justified scientifically
- Race/Ethnicity: Distribution justified scientifically
- Inclusion/Exclusion of Children under 21: Excluding ages < 21 justified scientifically
- 25% women, 50% African-American, children not included

Vertebrate Animals:

Not Applicable (No Vertebrate Animals)

Biohazards:

Not Applicable (No Biohazards)

Resubmission:

- The investigators have been reasonably responsive to reviewers concerns, though some limitations remain.

Budget and Period of Support:

Recommend as Requested

CRITIQUE 3:

Significance: 1
Investigator(s): 1
Innovation: 2
Approach: 2
Environment: 1

Overall Impact: This proposal tests two doses of Speed of Processing Training (10 hours vs. 20 hours) with an attention matched sham control condition to evaluate the efficacy of Speed of Processing on improving or delaying cognitive decline in people living and aging with HIV. This is a significant public health issue for which few behavioral interventions exist. This proposal is an extension of prior work from the current research team and builds upon strong preliminary research. The team has a great deal of expertise in this area, including developing the Speed of Processing training and testing it in a variety of populations and scenarios. The overall approach is sound and includes a two year follow-up period and a good retention plan. However, the role of ART and viral suppression/progression as either a covariate or outcome would be better articulated. The environment provides adequate resources to support the proposed project.

1. Significance:

Strengths

- Addresses neurocognitive decline in people living and aging with HIV—an important goal.
- The driving safety angle is an interesting and significant public health issue.
- The focus on the Southeastern US, which is highly impacted by HIV and also has significant resource allocation difficulties is significant.
- While ART has had a positive impact on HAND, this does remain a significant issue among people aging with HIV.

Weaknesses

- None.

2. Investigator(s):

Strengths

- Strong team with experience working together on similar projects.
- Complimentary areas of expertise appear adequate to demands of project.
- Study builds upon prior work of team and is informed by strong preliminary data.

Weaknesses

- None.

3. Innovation:

Strengths

- Behavioral interventions in improve cognitive functioning are needed.
- Current team pioneered techniques utilized in proposed intervention.
- Examines dosage (10 vs. 20 hours) of Speed of Processing training.

Weaknesses

- Several studies have now been completed using Speed of Processing Training.

4. Approach:

Strengths

- Three arm design with two doses of active intervention compared to a control condition is appropriate.
- Use of attention control sham condition is a strength.
- Broad inclusion is a strength.
- Two year follow-up with good retention plan.
- Comprehensive assessment battery—it is long, but this is typical of Neuropsychological testing. It could be possible to split assessment into two sessions, however.

Weaknesses

- Clinical data will be abstracted from medical records, which is useful, but more attention could be paid to ART adherence and viral load in regard to a potential covariate as well as a potential outcome, with cognitive processing as a mediator.

5. Environment:

Strengths

- Proposal includes a number of key partners (UAB CFAR, Roybal Center, School of Nursing, Psych Department) that contribute adequate support to proposed project.
- Similar projects have been completed in this setting in the past.

Weaknesses

- None

Protections for Human Subjects:

Acceptable Risks and/or Adequate Protections

- Plan for referring patients for care if depression or significant cognitive decline detected.

Data and Safety Monitoring Plan (Applicable for Clinical Trials Only):

Acceptable

- External panel of four to meet annually.

Inclusion of Women, Minorities and Children:

- Sex/Gender: Distribution justified scientifically
- Race/Ethnicity: Distribution justified scientifically
- Inclusion/Exclusion of Children under 21: Excluding ages < 21 justified scientifically
- Focus on adults aging with HIV, thus, including only age 40 and greater is justified. Sample will likely include race/ethnicity and gender representative of the area.

Vertebrate Animals:

Not Applicable (No Vertebrate Animals)

Biohazards:

Not Applicable (No Biohazards)

Resubmission:

- Proposal appears responsive to prior reviews and has replaced one prior study arm (home-based intervention) with an arm delivering a higher dose of the intervention. This appears to have strengthened the design and added an element of innovation. Additional design points have been further clarified.

Resource Sharing Plans:

Acceptable

- Plan is available and reasonable

Budget and Period of Support:

Recommend as Requested

THE FOLLOWING SECTIONS WERE PREPARED BY THE SCIENTIFIC REVIEW OFFICER TO SUMMARIZE THE OUTCOME OF DISCUSSIONS OF THE REVIEW COMMITTEE, OR REVIEWERS' WRITTEN CRITIQUES, ON THE FOLLOWING ISSUES:

PROTECTION OF HUMAN SUBJECTS (Resume): ACCEPTABLE

INCLUSION OF WOMEN PLAN (Resume): ACCEPTABLE

INCLUSION OF MINORITIES PLAN (Resume): ACCEPTABLE

INCLUSION OF CHILDREN PLAN (Resume): ACCEPTABLE

COMMITTEE BUDGET RECOMMENDATIONS: The budget was recommended as requested.

NIH has modified its policy regarding the receipt of resubmissions (amended applications). See Guide Notice NOT-OD-14-074 at http://grants.nih.gov/grants/guide/notice-files/NOT-OD-14-074.html. The impact/priority score is calculated after discussion of an application by averaging the overall scores (1-9) given by all voting reviewers on the committee and multiplying by 10. The criterion scores are submitted prior to the meeting by the individual reviewers assigned to an application, and are not discussed specifically at the review meeting or calculated into the overall impact score. Some applications also receive a percentile ranking. For

details on the review process, see http://grants.nih.gov/grants/peer_review_process.htm#scoring.

MEETING ROSTER

Behavioral and Social Consequences of HIV/AIDS Study Section
AIDS and Related Research Integrated Review Group
CENTER FOR SCIENTIFIC REVIEW
BSCH
November 05, 2015 - November 06, 2015

CHAIRPERSON

SEAL, DAVID W, PHD
PROFESSOR
DEPARTMENT OF GLOBAL COMMUNITY HEALTH
AND BEHAVIORAL SCIENCES
SCHOOL OF PUBLIC HEALTH AND TROPICAL MEDICINE
TULANE UNIVERSITY
NEW ORLEANS, LA 70112

MEMBERS

CHAMPION, JANE DIMMITT, PHD, DNP, FAAN *
PROFESSOR
SCHOOL OF NURSING
UNIVERSITY OF TEXAS AT AUSTIN
AUSTIN, TX 78701

COOK, ROBERT L, MPH, MD
PROFESSOR
DEPARTMENT OF EPIDEMIOLOGY
UNIVERSITY OF FLORIDA
GAINESVILLE, FL 32610

CORSI, KAREN F, MPH, SCD
ASSOCIATE PROFESSOR
DEPARTMENT OF PSYCHIATRY
SCHOOL OF MEDICINE
UNIVERSITY OF COLORADO, DENVER
DENVER, CO 80262

GIORDANO, THOMAS P, MPH, MD
ASSOCIATE PROFESSOR
DEPARTMENT OF MEDICINE
BAYLOR COLLEGE OF MEDICINE
HOUSTON, TX 77030

GWADZ, MARYA , PHD
SENIOR RESEARCH SCIENTIST
COLLEGE OF NURSING
NEW YORK UNIVERSITY
NEW YORK, NY 10003

HABERER, JESSICA ELIZABETH, MD *
ASSISTANT PROFESSOR OF MEDICINE
HARVARD INITIATIVE FOR GLOBAL HEALTH
MASSACHUSETTS GENERAL HOSPITAL
CAMBRIDGE, MA 02138

HANSEN, NATHAN B, PHD *
ASSOCIATE PROFESSOR AND DEPARTMENT HEAD
HEALTH PROMOTION AND BEHAVIOR
COLLEGE OF PUBLIC HEALTH
UNIVERSITY OF GEORGIA
ATLANTA, GA 30602

JOHNSON, MALLORY O, PHD *
PROFESSOR
DEPARTMENT OF MEDICINE
CENTER FOR AIDS PREVENTION STUDIES
UNIVERSITY OF CALIFORNIA, SAN FRANCISCO
SAN FRANCISCO, CA 94105

KERR, THOMAS , PHD
PROFESSOR
DEPARTMENT OF MEDICINE
BRITISH COLUMBIA CENTRE
FOR EXCELLENCE IN HIV/AIDS
UNIVERSITY OF BRITISH COLUMBIA
VANCOUVER, BC V6Z, 1Y6
CANADA

LI, XIAOMING , PHD
PROFESSOR
HEALTH PROMOTION, EDUCATION, AND BEHAVIOR
ARNOLD SCHOOL OF PUBLIC HEALTH
UNIVERSITY OF SOUTH CAROLINA
COLUMBIA, SC 29208

LOVEJOY, TRAVIS IAN, PHD *
ASSISTANT PROFESSOR
DEPARTMENT OF PSYCHIATRY
OREGON HEALTH AND SCIENCE UNIVERSITY
PORTLAND, OR 97239

MERCHANT, ROLAND C, MPH, SCD, MD
ASSOCIATE PROFESSOR
DEPARTMENT OF EMERGENCY MEDICINE
WARREN ALPERT MEDICAL SCHOOL
BROWN UNIVERSITY
PROVIDENCE, RI 02903

MIMIAGA, MATTHEW JAMES, SCD *
PROFESSOR
BEHAVIORAL AND SOCIAL SCIENCES AND
EPIDEMIOLOGY
SCHOOL OF PUBLIC HEALTH
BROWN UNIVERSITY
PROVIDENCE, RI 02903

NYAMATHI, ADELINE M, PHD
DISTINGUISHED PROFESSOR
SCHOOL OF NURSING
UNIVERSITY OF CALIFORNIA, LOS ANGELES
LOS ANGELES, CA 90095

OTTO-SALAJ, LAURA L, PHD *
ASSOCIATE PROFESSOR
DEPARTMENT OF SOCIAL WORK
HELEN BADER SCHOOL OF SOCIAL WELFARE
UNIVERSITY OF WISCONSIN, MILWAUKEE
MILWAUKEE, WI 53201

OWNBY, RAYMOND L, MD, PHD *
PROFESSOR AND CHAIR
DEPARTMENT OF PSYCHIATRY AND BEHAVIORAL
MEDICINE
COLLEGE OF OSTEOPATHIC MEDICINE
NOVA SOUTHEASTERN UNIVERSITY
FORT LAUDERDALE, FL 33314

PATTERSON, THOMAS L, PHD *
PROFESSOR
DEPARTMENT OF PSYCHIATRY
UNIVERSITY OF CALIFORNIA, SAN DIEGO
LA JOLLA, CA 92093

REYNOLDS, NANCY R, PHD
PROFESSOR
SCHOOL OF NURSING
YALE UNIVERSITY
NEW HEAVEN, CT 06536

RHODES, SCOTT D, PHD *
PROFESSOR AND CHAIR
DEPARTMENT OF SOCIAL SCIENCES AND HEALTH POLICY
WAKE FOREST UNIVERSITY SCHOOL OF MEDICINE
WINSTON-SALEM, NC 27157

SORENSEN, JAMES L, PHD
PROFESSOR
DEPARTMENT OF PSYCHIATRY
UNIVERSITY OF CALIFORNIA, SAN FRANCISCO
SAN FRANCISCO, CA 94110

STARRELS, JOANNA L, MD *
ASSOCIATE PROFESSOR
DIVISION OF GENERAL INTERNAL MEDICINE
ALBERT EINSTEIN COLLEGE OF MEDICINE
BRONX, NY 10467

STEPHENSON, ROBERT B, PHD *
PROFESSOR
SCHOOL OF NURSING
UNIVERSITY OF MICHIGAN
ANN ARBOR, MI 48109

TOBIN, KARIN E, PHD *
ASSOCIATE PROFESSOR
DEPARTMENT OF HEALTH, BEHAVIOR, AND SOCIETY
BLOOMBERG SCHOOL OF PUBLIC HEALTH
JOHNS HOPKINS UNIVERSITY
BALTIMORE, MD 21205

VASSILEVA, JASMIN L, PHD *
ASSOCIATE PROFESSOR
DEPARTMENT OF PSYCHIATRY
INSTITUTE FOR DRUG AND ALCOHOL STUDIES
VIRGINIA COMMONWEALTH UNIVERSITY
RICHMOND, VA 23219

VREEMAN, RACHEL CHRISTINE, MD
ASSOCIATE PROFESSOR
DEPARTMENT OF PEDIATRICS
SCHOOL OF MEDICINE
INDIANA UNIVERSITY
INDIANAPOLIS, IN 46202

WALDROP-VALVERDE, DRENNA , PHD
ASSOCIATE PROFESSOR
NELL HODGSON WOODRUFF SCHOOL OF NURSING
EMORY UNIVERSITY
ATLANTA, GA 30322

MAIL REVIEWER(S)

LI, JIANGHONG , MD
SENIOR RESEARCH SCIENTIST
INSTITUTE FOR COMMUNITY RESEARCH
HARTFORD, CT 06106

SIEGEL, KAROLYNN , PHD
PROFESSOR
DEPARTMENT OF SOCIOMEDICAL SCIENCES
COLUMBIA UNIVERSITY
MAILMAN SCHOOL OF PUBLIC HEALTH
NEW YORK, NY 10032

YARASHESKI, KEVIN E, PHD
PROFESSOR
DIVISION OF METABOLISM AND ENDOCRINOLOGY
DEPARTMENT OF MEDICINE
WASHINGTON UNIVERSITY SCHOOL OF MEDICINIE
ST LOUIS, MO 63110

SCIENTIFIC REVIEW OFFICER

RUBERT, MARK P, PHD
SCIENTIFIC REVIEW OFFICER
CENTER FOR SCIENTIFIC REVIEW
NATIONAL INSTITUTES OF HEALTH
BETHESDA, MD 20892

EXTRAMURAL SUPPORT ASSISTANT

BOGLEY, DAVID M
LEAD EXTRAMURAL SUPPORT ASSISTANT
CENTER FOR SCIENTIFIC REVIEW
NATIONAL INSTITUTES OF HEALTH
BETHESDA, MD 20892

* Temporary Member. For grant applications, temporary members may participate in the entire meeting or may review only selected applications as needed.

Consultants are required to absent themselves from the room during the review of any application if their presence would constitute or appear to constitute a conflict of interest.
